# Machine-learning-based prediction models for high-need high-cost patients using nationwide clinical and claims data

**DOI:** 10.1038/s41746-020-00354-8

**Published:** 2020-11-11

**Authors:** Itsuki Osawa, Tadahiro Goto, Yuji Yamamoto, Yusuke Tsugawa

**Affiliations:** 1 Department of Medicine, The University of Tokyo Hospital, Tokyo, Japan; 2 Department of Clinical Epidemiology and Health Economics, School of Public Health, The University of Tokyo, 7-3-1 HongoBunkyo-ku, Tokyo, 113-0033 Japan; 3 MinaCare Co., Ltd., Tokyo, Japan; 4 Division of General Internal Medicine and Health Service Research, David Geffen School of Medicine at UCLA, Los Angeles, CA USA; 5 Department of Health Policy and Management, UCLA Fielding School of Public Health, Los Angeles, CA USA

**Keywords:** Health care economics, Health policy

## Abstract

High-need, high-cost (HNHC) patients—usually defined as those who account for the top 5% of annual healthcare costs—use as much as half of the total healthcare costs. Accurately predicting future HNHC patients and designing targeted interventions for them has the potential to effectively control rapidly growing healthcare expenditures. To achieve this goal, we used a nationally representative random sample of the working-age population who underwent a screening program in Japan in 2013–2016, and developed five machine-learning-based prediction models for HNHC patients in the subsequent year. Predictors include demographics, blood pressure, laboratory tests (e.g., HbA1c, LDL-C, and AST), survey responses (e.g., smoking status, medications, and past medical history), and annual healthcare cost in the prior year. Our prediction models for HNHC patients combining clinical data from the national screening program with claims data showed a c-statistics of 0.84 (95%CI, 0.83–0.86), and overperformed traditional prediction models relying only on claims data.

## Introduction

Rapidly growing healthcare spending has become one of the significant challenges in many developed countries^1^. Existing evidence indicates that healthcare spending is concentrated among a small number of costly patients, known as high-need, high-cost (HNHC) patients—typically defined as those who account for the top 5% of annual healthcare costs. Research has shown that the top-1% and top-5% high-cost patients accounted for 23% and 50 %, respectively, of all healthcare costs^2^. A prediction model for future HNHC patients has been attracting the attention of policymakers and payers in recent years due to an expectation that interventions targeting this population may be more effective in reducing healthcare spending than interventions targeting the entire population^3–6^. Not only the Japanese government but also the Organization for Economic Co-operation and Development (OECD) is considering HNHC patients as one of the policy targets priorities that have the potential to effectively curb rapidly growing healthcare costs^7^. Therefore, a valid, reliable, and implementable approach to accurately predict HNHC patients in real-time is critically important for designing targeted interventions that can effectively lower healthcare spending.

Although studies have sought to develop accurate models for predicting HNHC patients, their performance remains suboptimal due to the complex interplay among predictors and the lack of detailed clinical information (e.g., body mass index [BMI], blood pressure level, laboratory data) in the data used to construct the prediction models. Many of the existing studies on prediction models of high-cost patients relied on data from claims, self-reported data, or electronic health records that do not include laboratory test results^8–17^. Evidence is limited as to whether the data from laboratory tests—which are, arguably, more granular and detailed clinical information—leads to the improvement of the performance of the prediction model. A recent study using data from the health screening program and claims data in South Korea reported an improvement in the performance of the machine-learning-based prediction model for the top 10% of high-cost patients^18^. However, despite that many insurers and providers globally are actively seeking for an approach that can accurately predict HNHC patients^3–6,13,19,20^, it remains largely unclear whether the machine-learning-based prediction model using the detailed clinical information collected through a health screening program combined with claims data could achieve high prognostic performance for predicting HNHC patients in subsequent years^18^.

In this context, we developed and evaluated machine-learning-based prediction models for HNHC patients using data from national screening programs and claims in Japan. A developed model based on administrative data would be immensely helpful for policymakers and payers to identify effective strategies to contain rapidly growing healthcare costs.

## Results

### Beneficiary characteristics

**Fig. 1:**
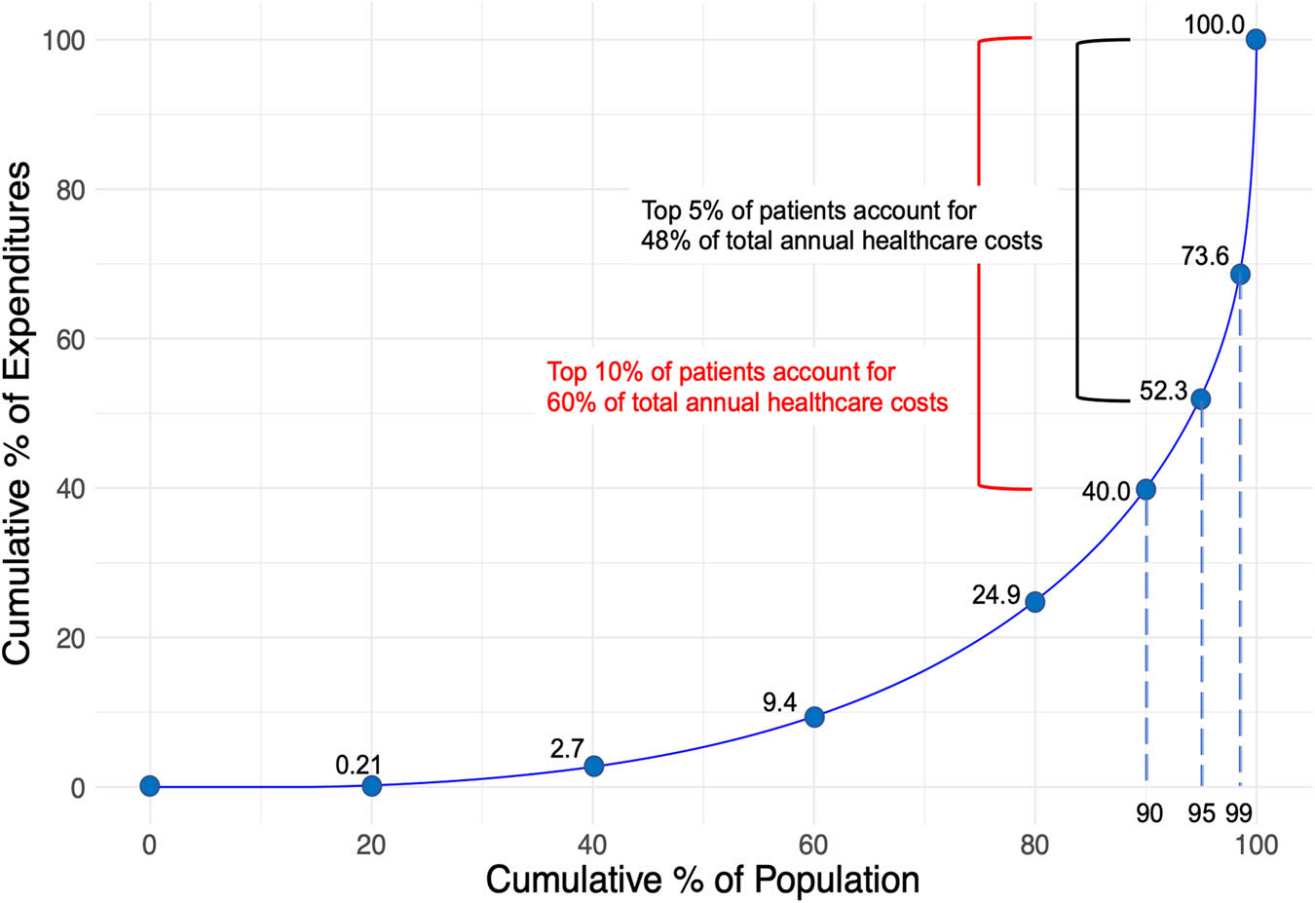
Distribution of annual healthcare costs in the working-age population in Japan, 2016. In 2016, the top 1%, 5%, 10% of patients accounted for 26.4%, 47.7%, and 60.0% of total annual healthcare costs.

During the study period, the database included 363,165 adults who underwent the national screening programs every fiscal year (from April 1 through March 31) in 2013–2016. Of those, we used a 10% random sample (*n* = 36,316) for the analyses (Table 1). Among 36,316 individuals in our analytic cohort, 21,985 (61%) were male, and the median age in 2013 was 43 years. In 2016, the median annual healthcare cost was 43,270 JPY (376 USD; using an exchange rate of 115 yen per US dollar as of December 2016), and the top 1%, 5%, 10% of patients accounted for 26%, 48%, and 60%, respectively, of all amounts of annual healthcare costs (Fig. 1).

**Table 1:** Predictor variables and outcome in 36,316 people.

Variables	In 2013	In 2014	In 2015	In 2016
Age (year), median (IQR)	43 (37–50)	44 (38–51)	45 (39–52)	–
Male gender	21,985 (61)	21,985 (61)	21,985 (61)	–
Height (cm)	167 (160–173)	167 (160–173)	167 (160–173)	–
Body weight (kg)	63 (54–72)	63 (54–72)	63 (54–72)	–
Waist circumference (cm)	81 (75–87)	81 (74–87)	81 (75–88)	–
Vital signs				
Systolic blood pressure (mmHg), median (IQR)	116 (106–127)	117 (106–127)	117 (106–127)	–
Diastolic blood pressure (mmHg), median (IQR)	72 (65–81)	73 (65–81)	73 (65–81)	–
Laboratory data				
Fasting blood sugar (mg/dl), median (IQR)	91 (85–97)	91 (86–98)	91 (86–98)	–
HbA1c (%), median (IQR)	5.4 (5.2–5.6)	5.4 (5.2–5.6)	5.4 (5.2–5.7)	–
TG (mg/dl), median (IQR)	85 (59–129)	85 (60–129)	86 (60–129)	–
LDL-C (mg/dl), median (IQR)	119 (98–141)	119 (99–141)	120 (100–142)	–
HDL-C (mg/dl), median (IQR)	59 (50–71)	60 (50–72)	60 (50–73)	–
AST (IU/l), median (IQR)	20 (17–24)	20 (17–24)	20 (17–25)	–
ALT (IU/l), median (IQR)	18 (13–27)	18 (13–27)	18 (13–27)	–
y-GTP (IU/l), median (IQR)	24 (17–42)	25 (17–42)	25 (17–42)	–
ECG abnormalities	362 (1)	611 (2)	632 (2)	–
Survey responses				
Medications				
Anti-hypertensive drugs	2,776 (8)	2,975 (8)	3,207 (9)	–
Hypoglycemic drugs	738 (2)	801 (2)	895 (2)	–
Anti-hyperlipidemic drugs	1697 (5)	1803 (5)	1,990 (5)	–
Past medical history				
Stroke	143 (0.4)	231 (0.6)	224 (0.6)	–
Cardiovascular diseases	271 (0.7)	417 (1)	471 (1)	–
Kidney diseases	38 (0.1)	55 (0.2)	36 (0.1)	–
Current smoking	8299 (23)	8260 (23)	8158 (22)	–
Exercise > 30 min twice a week for a year	4301 (12)	5667 (16)	5,919 (16)	–
Annual healthcare cost (JPY) [USD], median (IQR)	32,930 [286] (8040–100,710)	36,830 [320] (9270–108,300)	41,860 [364] (10,740–108,300)	43,270 [376] (11,390–121,390)

Values represent *n* (%), unless otherwise indicated.

*IQR* interquartile range, *HbA1c* hemoglobin A1c, TG triglycerides, *LDL-C* low-density lipoprotein cholesterol, *HDL-C* high-density lipoprotein cholesterol, *AST* aspartate aminotransferase, *ALT* alanine aminotransferase, *y-GTP* gamma-glutamyl transpeptidase, *ECG* electrocardiogram.

### Prediction of HNHC patients

**Fig. 2:**
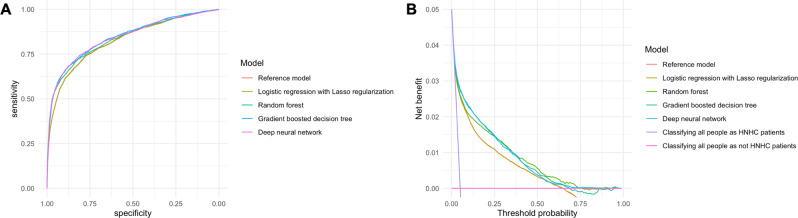
Prediction ability of the reference and machine-learning-based prediction models for HNHC patients. **A** Receiver-operating-characteristics (ROC) curves. The corresponding values of the area under the receiver-operating-characteristics curve for each model (i.e., the c-statistics) are presented in Table 2. **B** Decision curve analysis. The *X*-axis indicates the threshold probability for HNHC patients. The *Y*-axis indicates the net benefit. The curves (decision curves) indicate the net benefit of models (the reference model and four machine-learning-based models) as well as two clinical alternatives (classifying no people as HNHC patients vs. classifying all people as HNHC patients) over a specified range of threshold probabilities of outcome. Compared to the reference model, the net benefit, which is defined as the following equation, for all machine-learning-based models was greater across the range of threshold probabilities. “net benefit = (1 − false negative) × prevalence − false positive × (1 − prevalence) × the odds at the threshold probability”. HNHC patients = high-need, high-cost patients.

The discrimination ability of different models, as represented by ROC curves, is shown in Fig. 2. The logistic regression model (the reference model) had the lowest discriminative ability (c-statistics, 0.82; 95%CI, 0.81–0.84), while all other machine-learning-based models had a high discriminative ability (Table 2). For example, the gradient-boosting decision tree model had significantly higher c-statistics (0.84, 95%CI, 0.83–0.86; *P* = 0.01) compared to the reference model. The reference model had the highest sensitivity (0.74; 95%CI, 0.72–0.76), and the gradient-boosting decision tree model had the highest specificity (0.88; 95%CI, 0.87–0.88). Considering the low prevalence of outcome (5%), the highest positive predictive value was 0.22 (95%CI, 0.22–0.24) in the gradient-boosting decision tree model, and the negative predictive values were all high (0.98 [95%CI, 0.98–0.98] in all models). The gradient-boosting decision tree model had the highest positive likelihood ratio of 5.5 (95%CI, 5.3–5.7), and the reference model had the best negative likelihood ratio of 0.34 (95%CI, 0.31–0.36). In the decision curve analysis (Fig. 2), compared with the reference and the Lasso regression model, the net benefit for other machine-learning-based models (e.g., the random forest model) was higher over the range of threshold probabilities, with the random forest and gradient-boosted decision tree model having the greatest net benefit. Given that the number of HNHC patients in our cohort (1815 adults) was substantially larger than the number of parameters used for the prediction (25 parameters), we assumed that the risk of overfitting was low. Indeed, the event per variable (EPV) for our primary prediction model was 72, indicating a low risk of overfitting (EPV < 20 is indicative of potential overfitting)^21^. We found no predictors with high variance inflation factors (VIF) (>10) among parameters included in our reference model, indicating that collinearity is not an issue for our prediction models (Supplementary Table 1)^22^.

**Table 2:** Prediction ability of the reference and four machine-learning-based prediction models for HNHC patients.

Outcome	c-statistics	*P*-value^b^	Sensitivity	Specificity	PPV	NPV	PLR	NLR
Reference model^a^	0.82 (0.81–0.84)	[Reference]	0.74 (0.72–0.76)	0.78 (0.77–0.78)	0.15 (0.14–0.16)	0.98 (0.98–0.98)	3.3 (3.2–3.4)	0.34 (0.31–0.36)
Logistic regression with Lasso regularization	0.82 (0.81–0.84)	0.98	0.67 (0.65–0.79)	0.85 (0.84–0.85)	0.19 (0.18–0.20)	0.98 (0.98–0.98)	4.3 (4.2–4.5)	0.39 (0.37–0.42)
Random forest	0.84 (0.83–0.85)	0.12	0.71 (0.69–0.73)	0.83 (0.83–0.84)	0.18 (0.17–0.19)	0.98 (0.98–0.98)	4.2 (4.1–4.4)	0.35 (0.32–0.37)
Gradient-boosted decision tree	0.84 (0.83–0.86)	0.01	0.68 (0.66–0.71)	0.88 (0.87–0.88)	0.22 (0.21–0.24)	0.98 (0.98–0.98)	5.5 (5.3–5.7)	0.36 (0.34–0.39)
Deep neural network	0.84 (0.83–0.85)	0.03	0.71 (0.68–0.73)	0.85 (0.85–0.86)	0.20 (0.19–0.21)	0.98 (0.98–0.98)	4.8 (4.6–5.0)	0.35 (0.32–0.37)

*HNHC* high-need, high-cost, *PPV* positive predictive value, *NPV* negative predictive value, *PLR* positive likelihood ratio, *NLR* negative likelihood ratio.

^a^We used a non-penalized logistic regression model as the reference model.

^b^We compared the area under the curve between each machine-learning-based prediction model and the logistic regression model (the reference model) using the DeLong’s test.

### Variable importance

**Fig. 3:**
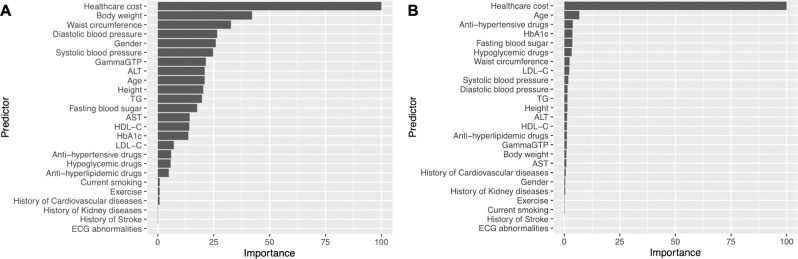
Importance of each predictor in the random forest and gradient-boosted decision tree model. **A** The Random forest model. **B** The Gradient-boosted decision tree model. The variable importance is a measure scaled to have a maximum value of 100. HbA1c hemoglobin A1c, TG triglycerides, LDL-C low-density lipoprotein cholesterol, HDL-C high-density lipoprotein cholesterol, AST aspartate aminotransferase, ALT alanine aminotransferase, GammaGTP gamma-glutamyl transpeptidase, ECG electrocardiogram.

The variable importance in the random forest and gradient-boosted decision tree model (model 1) was demonstrated in Fig. 3. In both models, healthcare cost in the previous year was the most important predictor for HNHC patients. In the random forest model, obesity-related metrics (e.g., body weight and waist circumference), gender, and blood pressure were significant in addition to healthcare cost. In the gradient-boosted decision tree model, age, the use of anti-hypertensive drugs, and predictors related to blood sugar level (e.g., Hb1Ac, fasting blood sugar, and hypoglycemic drugs) were important in addition to healthcare cost.

### Sensitivity analyses

We found a similar high performance of the machine-learning-based prediction models when we used different thresholds for defining HNHC patients (Table 3). The Lasso regression model had the highest prediction performance (c-statistics, 0.86; 95%CI, 0.84–0.88) for predicting those who account for the top 1% of healthcare cost, and the gradient-boosted decision tree model had the highest prediction performance (c-statistics, 0.88; 95%CI, 0.87–0.88) for predicting those who account for the top 10% of healthcare cost. We found no qualitative differences in the discriminative ability between the prediction models using single-year data and those using consecutive 2-year data (Tables 4 and 5). Annual healthcare cost in the previous year was the most important predictor for HNHC patients in the subsequent year (Fig. 3). The machine-learning-based prediction models using both clinical data from the screening program and healthcare cost calculated using claims data marginally improved the prediction performance compared to the models using only patient age, gender, and healthcare cost calculated using claims data (Tables 6 and 7). We found that adding 21 major diagnosis categories and 22 major procedure categories to a set of predictors included in our model did not improve the prediction ability significantly compared with the reference model (i.e., model using the healthcare cost data) (e.g., c-statistics [95% CI] of random forest model, 0.83 [0.82–0.85] vs. 0.84 [0.83–0.85]; Supplementary Table 2).

**Table 3:** Prediction ability of the reference and four machine-learning-based prediction models for top 1% or 10% healthcare cost spenders.

Outcome	c-statistics	*P-*value^b^	Sensitivity	Specificity	PPV	NPV	PLR	NLR
The prediction model for top 1% healthcare cost spenders								
Reference model^a^	0.85 (0.82–0.87)	[Reference]	0.71 (0.67–0.76)	0.84 (0.83–0.84)	0.04 (0.04–0.05)	0.99 (0.99–0.99)	4.4 (4.1–4.7)	0.35 (0.29–0.41)
Logistic regression with Lasso regularization	0.86 (0.84–0.88)	0.42	0.78 (0.73–0.82)	0.78 (0.78–0.79)	0.04 (0.03–0.04)	0.99 (0.99–0.99)	3.6 (3.4–3.8)	0.29 (0.24–0.35)
Random forest	0.83 (0.80–0.85)	0.26	0.66 (0.61–0.71)	0.88 (0.87–0.88)	0.05 (0.05–0.06)	0.99 (0.99–0.99)	5.4 (4.9–5.8)	0.39 (0.34–0.45)
Gradient-boosted decision tree	0.85 (0.83–0.88)	0.69	0.70 (0.65–0.74)	0.87 (0.87–0.88)	0.05 (0.05–0.06)	0.99 (0.99–0.99)	5.4 (5.0–5.8)	0.35 (0.30–0.41)
Deep neural network	0.85 (0.82–0.87)	0.91	0.74 (0.69–0.78)	0.80 (0.80–0.80)	0.04 (0.03–0.04)	0.99 (0.99–0.99)	3.7 (3.4–3.9)	0.33 (0.28–0.39)
The prediction model for top 10% healthcare cost spenders								
Reference model^a^	0.85 (0.85–0.86)	[Reference]	0.74 (0.73–0.76)	0.83 (0.83–0.84)	0.33 (0.32–0.34)	0.97 (0.97–0.97)	4.4 (4.3–4.6)	0.31 (0.29–0.33)
Logistic regression with Lasso regularization	0.85 (0.85–0.86)	0.99	0.74 (0.73–0.76)	0.83 (0.83–0.84)	0.33 (0.32–0.34)	0.97 (0.97–0.97)	4.5 (4.3–4.6)	0.31 (0.30–0.33)
Random forest	0.87 (0.86–0.88)	<0.001	0.75 (0.73–0.76)	0.87 (0.87–0.88)	0.39 (0.38–0.41)	0.97 (0.97–0.97)	5.8 (5.7–6.1)	0.29 (0.27–0.31)
Gradient-boosted decision tree	0.88 (0.87–0.88)	<0.001	0.76 (0.75–0.77)	0.87 (0.86–0.87)	0.38 (0.37–0.40)	0.97 (0.97–0.97)	5.6 (5.4–5.8)	0.28 (0.26–0.30)
Deep neural network	0.88 (0.87–0.88)	<0.001	0.75 (0.74–0.77)	0.87 (0.87–0.88)	0.39 (0.38–0.41)	0.97 (0.97–0.97)	5.8 (5.6–6.0)	0.28 (0.27–0.30)

*PPV* positive predictive value, *NPV* negative predictive value, *PLR* positive likelihood ratio, *NLR* negative likelihood ratio.

^a^We used a non-penalized logistic regression model as the reference model.

^b^We compared the area under the curve between each machine-learning-based prediction model and the logistic regression model (the reference model) using the DeLong’s test.

**Table 4:** Prediction ability of the reference and four machine learning models using consecutive 2-year data for HNHC patients.

Outcome	c-statistics	*P*-value^b^	Sensitivity	Specificity	PPV	NPV	PLR	NLR
Reference model^a^	0.83 (0.82–0.84)	[Reference]	0.67 (0.64–0.69)	0.87 (0.86–0.87)	0.21 (0.20–0.22)	0.98 (0.98–0.98)	4.9 (4.7–5.1)	0.39 (0.36–0.41)
Logistic regression with Lasso regularization	0.83 (0.82–0.84)	0.93	0.69 (0.67–0.71)	0.85 (0.84–0.85)	0.19 (0.18–0.20)	0.98 (0.98–0.98)	4.5 (4.3–4.7)	0.37 (0.35–0.40)
Random forest	0.84 (0.83–0.85)	0.22	0.66 (0.64–0.68)	0.88 (0.88–0.89)	0.23 (0.22–0.24)	0.98 (0.98–0.98)	5.6 (5.4–5.9)	0.39 (0.36–0.41)
Gradient-boosted decision tree	0.84 (0.83–0.86)	0.05	0.68 (0.66–0.71)	0.88 (0.87–0.88)	0.22 (0.21–0.24)	0.98 (0.98–0.98)	5.5 (5.3–5.7)	0.36 (0.34–0.39)
Deep neural network	0.84 (0.83–0.85)	0.18	0.69 (0.67–0.71)	0.86 (0.86–0.87)	0.21 (0.20–0.22)	0.98 (0.98–0.98)	5.0 (4.8–5.2)	0.36 (0.34–0.39)

*PPV* positive predictive value, *NPV* negative predictive value, *PLR* positive likelihood ratio, *NLR* negative likelihood ratio.

^a^We used a non-penalized logistic regression model as the reference model.

^b^We compared the area under the curve between each machine-learning-based prediction model and the logistic regression model (the reference model) using the DeLong’s test.

**Table 5:** Comparison of the prediction ability for HNHC patients between the model using single-year and consecutive 2-year data.

	c-statistics		*P*-value^b^
	Using single-year data	Using consecutive 2-year data	
Reference model^a^	0.824 (0.813–0.835)	0.829 (0.818–0.840)	0.56
Logistic regression with Lasso regularization	0.824 (0.813–0.835)	0.830 (0.818–0.841)	0.52
Random forest	0.837 (0.826–0.848)	0.839 (0.828–0.850)	0.80
Gradient-boosted decision tree	0.844 (0.833–0.855)	0.845 (0.834–0.856)	0.96
Deep neural network	0.842 (0.831–0.853)	0.840 (0.828–0.851)	0.81

^a^We used a non-penalized logistic regression model as the reference model.

^b^We compared the area under the curve between each machine-learning-based prediction model and the logistic regression model (the reference model) using the DeLong’s test.

**Table 6:** Prediction ability of the reference and four machine-learning-based prediction models for HNHC patients using part of clinical and claims data.

Outcome	c-statistics	*P*-value^b^	Sensitivity	Specificity	PPV	NPV	PLR	NLR
The prediction model using only clinical data collected from the screening program								
Reference model^a^	0.71 (0.70–0.72)	[Reference]	0.56 (0.54–0.58)	0.75 (0.75–0.75)	0.11 (0.10–0.11)	0.97 (0.97–0.97)	2.2 (2.1–2.3)	0.59 (0.56–0.62)
Logistic regression with Lasso regularization	0.71 (0.70–0.72)	0.99	0.54 (0.52–0.56)	0.77 (0.77–0.78)	0.11 (0.10–0.12)	0.97 (0.97–0.97)	2.4 (2.3–2.5)	0.60 (0.57–0.63)
Random forest	0.74 (0.73–0.75)	0.001	0.64 (0.62–0.66)	0.71 (0.71–0.72)	0.11 (0.10–0.11)	0.97 (0.97–0.98)	2.2 (2.1–2.3)	0.51 (0.48–0.54)
Gradient-boosted decision tree	0.72 (0.70–0.73)	0.41	0.62 (0.60–0.65)	0.70 (0.69–0.70)	0.10 (0.09–0.10)	0.97 (0.97–0.97)	2.1 (2.0–2.1)	0.54 (0.51–0.57)
Deep neural network	0.72 (0.70–0.73)	0.39	0.53 (0.51–0.56)	0.79 (0.79–0.80)	0.12 (0.11–0.13)	0.97 (0.97–0.97)	2.6 (2.4–2.7)	0.59 (0.56–0.62)
The prediction model using only patient age, gender, and healthcare cost data from claims data								
Reference model^a^	0.82 (0.81–0.83)	[Reference]	0.68 (0.66–0.70)	0.84 (0.84–0.84)	0.18 (0.17–0.19)	0.98 (0.98–0.98)	4.2 (4.1–4.4)	0.38 (0.36–0.41)
Logistic regression with Lasso regularization	0.82 (0.81–0.83)	0.99	0.68 (0.66–0.70)	0.84 (0.84–0.85)	0.18 (0.18–0.19)	0.98 (0.98–0.98)	4.3 (4.1–4.5)	0.38 (0.36–0.41)
Random forest	0.82 (0.80–0.83)	0.53	0.63 (0.61–0.65)	0.88 (0.87–0.88)	0.21 (0.20–0.22)	0.98 (0.98–0.98)	5.1 (4.9–5.4)	0.42 (0.40–0.45)
Gradient-boosted decision tree	0.84 (0.83–0.85)	0.02	0.67 (0.64–0.69)	0.89 (0.89–0.89)	0.24 (0.23–0.25)	0.98 (0.98–0.98)	6.0 (5.7–6.2)	0.38 (0.35–0.40)
Deep neural network	0.84 (0.83–0.85)	0.02	0.69 (0.67–0.72)	0.86 (0.86–0.87)	0.21 (0.20–0.22)	0.98 (0.98–0.98)	5.1 (4.9–5.3)	0.35 (0.33–0.38)

*PPV* positive predictive value, *NPV* negative predictive value, *PLR* positive likelihood ratio, *NLR* negative likelihood ratio.

^a^We used a non-penalized logistic regression model as the reference model.

^b^We compared the area under the curve between each machine-learning-based prediction model and the logistic regression model (the reference model) using the DeLong’s test.

**Table 7:** Contribution of the predictors to the prediction ability.

	c-statistics		*P*-value^b^	c-statistics		*P*-value^b^
	Using clinical data and healthcare cost	Using only clinical data		Using clinical data and healthcare cost	Using patient age, gender, and healthcare cost	
Reference model^a^	0.824 (0.813–0.835)	0.708 (0.695–0.721)	<0.001	0.824 (0.813–0.835)	0.821 (0.809–0.833)	0.72
Logistic regression with Lasso regularization	0.824 (0.813–0.835)	0.708 (0.695–0.721)	<0.001	0.824 (0.813–0.835)	0.821 (0.809–0.833)	0.69
Random forest	0.837 (0.826–0.848)	0.738 (0.725–0.751)	<0.001	0.837 (0.826–0.848)	0.816 (0.804–0.828)	0.01
Gradient-boosted decision tree	0.844 (0.833–0.855)	0.716 (0.703–0.728)	<0.001	0.844 (0.833–0.855)	0.841 (0.830–0.852)	0.66
Deep neural network	0.842 (0.831–0.853)	0.716 (0.703–0.729)	<0.001	0.842 (0.831–0.853)	0.839 (0.828–0.851)	0.63

^a^We used a non-penalized logistic regression model as the reference model.

^b^We compared the area under the curve between each machine-learning-based prediction model and the logistic regression model (the reference model) using the DeLong’s test.

## Discussion

Using the nationally representative data of individuals who underwent the national screening program in Japan, we found that HNHC patients accounted for almost half of all annual healthcare costs, similar to the findings from other developed countries^2,4,13^. Machine-learning-based prediction models using both clinical data and healthcare cost calculated using claims data exhibited good prognostic performance for predicting HNHC patients in the subsequent year, compared with the prediction models relying only on claims data. The prediction models using consecutive 2-year data did not significantly improve the prediction performance compared to the models using single-year data. Taken together, these findings highlight the importance of incorporating clinical data—such as laboratory test results—in developing machine-learning to achieve high performance in predicting HNHC patients.

We found that adding clinical data from the screening program to the data on healthcare cost from claims marginally improved the performance of the prediction models. On the other hand, we found no meaningful improvements in prediction ability by adding the extra information on diagnosis and procedure to our prediction models—the finding consistent with prior studies^12,18^. This is probably because healthcare cost is a function of the individual billing codes (e.g., the diagnosis, procedures, and medications billed during hospitalization or the outpatient visit) available in the claims data, and therefore, once the healthcare cost in the preceding year is included as a predictor, the additional benefit of adding a broad set of variables from the claims may be negligible. On the contrary, clinical data from the national screening program used in our study may provide complementary information about the participants’ health status that are not available in the claims data (e.g., HbA1c level), and thus, the inclusion of both clinical data from the screening program and healthcare cost data from claims led to a better performance of the prediction model compared to the models that used only one of these two databases.

Multiple studies to date have sought to identify future HNHC patients using conventional approaches such as logistic regression models^9,11,12,14^. For example, a study that developed a model using logistic regression to predict the top 25% of HNHC patients of employees among the United Auto Workers using claims data and self-reported health data reported the c-statistics of 0.78 and 0.73, respectively^14^. While these conventional approaches had moderate prognostic performance, advanced machine-learning-based approaches have the potential to improve their prediction ability^8,12,15,18^ and possess scalability (e.g., extracting important features from a prediction model using many variables without physicians’ interpretations)^23^. Tamang et al. developed a model using elastic-net penalized logistic regression to predict the top 10% of HNHC patients using claims data in Denmark and showed the best c-statistics of 0.84^12^. The prediction ability of our prediction model for HNHC patients was better than similar machine-learning-based prediction models reported in previous studies^12,16^. The difference is likely due to the inclusion of detailed clinical data collected through the screening program. Until recently, there have been some studies that developed a machine-learning-based prediction model for HNHC patients using clinical data from administrative claims data^8–17^; however, few studies developed prediction models incorporating laboratory data to claims data. In 2019, Kim and colleagues analyzed the data from South Korea and found a marginal improvement in prediction ability for the top 10% of HNHC patients by adding clinical data from the screening program to claims data^18^. Our findings using Japan’s data were consistent with what they found using South Korean data, which supports the robustness and generalizability of our findings.

Our study has limitations. First, our data mainly consisted of the working-age population aged 18–75 years who underwent the national screening program. Therefore, our findings may not be generalizable to other age groups such as children and the elderly. This is particularly important as elderly people often account for a large proportion of healthcare expenditures in developed countries^24,25^. Second, our data from the national screening program included missing data (0.1%–36% of data in continuous variables), which could be a potential source of bias. However, we believed this issue could be minimized in our analyses under the use of random forest imputation for missing continuous variables (known to be a rigorous technique for the imputation of missing data)^26^. Lastly, given that we used nationwide data from Japan, our findings may not be generalized to the prediction of HNHC patients in other countries. However, our findings were consistent with a recent study conducted in South Korea^18^, which suggests potentially high generalizability of our findings in other contexts.

In summary, using nationally representative data from Japan, machine-learning-based models for predicting HNHC patients using clinical data from the national screening program and claims yielded a good prediction performance. In Japan, the time between the submission of claims by healthcare providers to insurers and the data to be available for the analysis is approximal two months. Therefore, our prediction models have the potential to inform policymakers and insurers by accurately predicting future HNHC patients in real-time and intervene if necessary, with the aim of curbing rapidly growing healthcare costs due to the aging population.

## Methods

### Data source and study population

We analyzed data from the nationwide claims database (MinaCare database) from April 1, 2013, to March 31, 2016. The MinaCare database collects claims from large employers and currently covers ~7.3% of the Japanese working population. This database includes working individuals and their dependent family members, with a wide range of age groups^27^. From the nationwide claims database, we used a 10% random sample of 363,165 adults aged 18 years and older who underwent the national screening programs every year from 2013 through 2016 (~45% of participants who received the screening in 2013 were included in our final sample).

In Japan, all adults are required by law to undergo the national screening program at least once a year, according to the Industrial Safety and Health Act enacted in 1972^28^. The screening program is standardized nationally, and includes several examinations, tests, and surveys, including demographics (height, body weight, waist circumference), eyesight, hearing, chest X-rays, blood pressure, laboratory tests (blood tests and urinalysis), electrocardiograms, past medical history, occupational history, and subjective and objective symptoms (Supplementary Table 3).

### Candidate predictors

We selected the candidate predictors from a broad set of variables based on our clinical knowledge. Specifically, from the national screening program data, we selected patient demographics (age, gender, body size measurements [height, body weight, waist circumference], systolic and diastolic blood pressure levels, laboratory data (fasting blood glucose, hemoglobin A1c [HbA1c], triglyceride [TG], high-density lipoprotein cholesterol [HDL-C], low-density lipoprotein cholesterol [LDL-C], aspartate aminotransferase [AST], alanine aminotransferase [ALT], gamma-glutamyl transpeptidase [γGTP]), electrocardiogram [ECG] abnormalities, and survey responses (medication use [anti-hypertensive, hypoglycemic and anti-hyperlipidemic drugs], past medical history [stroke, cardiovascular diseases, kidney diseases], current smoking, exercise status [>30 min twice a week for 1 year]). From the claims data, we included annual healthcare cost in the prior year, which includes all healthcare costs (except for a very small proportion of healthcare services that were not covered by health insurance [e.g., costs for cosmetic surgeries, the costs of over-the-counter drugs]) and has been shown to be one of the strongest predictors of future healthcare costs^7,15,18^. We decided to use only the data on healthcare cost in prior years the development of our primary prediction models because prior studies found no meaningful improvements in the prediction ability by adding a large number of variables available in the claims data (e.g., the diagnosis, procedures, and medications billed during hospitalization or the outpatient visit) to the data on healthcare cost^12,18^.

### Outcomes

The outcome was becoming an HNHC patient in the subsequent year. We defined HNHC patients as those who account for the top 5% of annual healthcare costs, an approach used in prior studies^4,6^.

### Statistical analysis

#### Machine-learning-based models

We developed five machine-learning-based models to predict HNHC patients in the subsequent year: (1) logistic regression (used as the reference model), (2) logistic regression with Lasso regularization (Lasso regression)^29^, (3) random forest^30^, (4) gradient-boosted decision tree^31^, and (5) deep neural network^32^. Lasso regularization is an extended standard regression model with the regularization parameter (lambda) to shrink large coefficients toward zero and minimize potential overfitting in the model by using a *glmnet* package^29,33^. Random forest is an ensemble of decision trees created by bootstrap aggregation and random feature selection^34^. Gradient-boosted decision tree is an additive model of decision trees estimated by gradient descent^31,35^. We used a grid search strategy to identify the best tuning hyperparameters by using *ranger* and *caret* packages for the random forest and gradient-boosted decision tree model^30,36^. Deep neural network is a machine-learning algorithm using multiple layers to model the nonlinear relationship between predictors and outcome^37^. We constructed a multiple-layer, feedforward model with adaptive moment estimation optimizer^38^ using a *keras* package for R version 3.6.1^32^ and developed the final models by manual tuning of the hyperparameters (i.e., the number of layers, hidden units, learning rate, learning rate decay, dropout rate, batch size, and epochs).

#### Model development, validation, and assessment

We first developed prediction models using predictors in the 2014 data and the outcome in the 2015 data (i.e., HNHC patients in 2015). Next, we validated these prediction models using predictors in the 2015 data and the outcome in the 2016 data. All predictors we used and the number of missing and non-responded data are shown in Supplementary Table 4. We conducted multiple imputations for missing data in continuous variables by using the random forest method^39^, and used the following variables for multiple imputations: patient demographics, blood pressure levels, laboratory data, and survey responses. Random forest imputation is a nonparametric algorithm that can accommodate nonlinearities and interactions and does not require a particular parametric model to be specified^26^. The single point estimates were generated by random draws from independent normal distributions centered on conditional means predicted using random forest. Random forest uses bootstrap aggregation of multiple regression trees to reduce the risk of overfitting, and it combines the estimates from many trees^39^. On the contrary, all non-responded data in survey responses (i.e., questionnaires for past medical history, social history) and ECG abnormalities, were assumed to be normal based on clinical reasoning. In model development and validation, we used several techniques to minimize potential overfitting—e.g., (1) Lasso regularization, (2) cross-validation (Lasso regularization, random forest, and gradient-boosted decision tree), (3) out-of-bag estimation (random forest, and gradient-boosted decision tree), (4) dropout and batch normalization (artificial neural network), and (5) validation of each model by using the data in different years. To address the potential collinearity of parameters included in our prediction model, we calculated the variance inflation factor (VIF)^22^.

The prediction performance of each model was assessed by computing (1) c-statistics (i.e., the area under the receiver-operating-characteristics [ROC] curve), (2) prospective prediction results (i.e., sensitivity, specificity, positive predictive value, negative predictive value, positive likelihood ratio, and negative likelihood ratio), and (3) decision curve analysis. To address the class imbalance in the outcome (e.g., the low proportion of individuals who were classified as HNHC), we chose the threshold of prospective prediction results based on the ROC curve (i.e., the Youden index)^40^. The decision curve analysis is a measure that takes into account the different weights of different misclassification types with a direct clinical interpretation (e.g., trade-offs between under- and over-estimation for each model)^41,42^. Specifically, the relative impact of false-negative (under-estimation) and false-positive (over-estimation) results given a threshold probability (or clinical preference) was accounted to yield a “net benefit” in each model. The net benefit of each model over a specified range of threshold probabilities of outcome was defined as “Eq. (1)” and graphically displayed as a decision curve^41,42^.

1 $$\begin{array}{l}{\mathrm{net}}\,{\mathrm{benefit}} = \left( {1 - {\mathrm{false}}\,{\mathrm{negative}}} \right) \times {\mathrm{prevalence}}\\ \begin{array}{*{20}{c}} { - {\mathrm{false}}\,{\mathrm{positive}} \times \left( {1 - {\mathrm{prevalence}}} \right) \times {\mathrm{the}}\,{\mathrm{odds}}\,{\mathrm{at}}\,{\mathrm{the}}\,{\mathrm{threshold}}\,{\mathrm{probability}}} \end{array}\end{array}$$

To gain insights into the contribution of each predictor to machine-learning-based models, we also computed the variable importance in the random forest and the gradient-boosted decision tree. The variable importance is a scaled measure to have a maximum value of 100^36,43^. DeLong’s test was used to compare ROC curves^44^.

### Sensitivity analyses

We conducted several sensitivity analyses. First, we used different thresholds for defining HNHC patients: those who account for the (1) top 1% and (2) top 10% of annual healthcare costs. Second, as the prediction models using longitudinal data may have better prediction ability, we developed prediction models using consecutive two-year data (i.e., data in 2013–2014) and the outcome in 2015. We then validated the models using predictors in 2014–2015 and the outcome in 2016. To assess the benefit of including clinical data from the national screening programs as predictors, we compared three machine-learning-based prediction models: (1) the model using only clinical data collected through the screening program, (2) the model only using patient age, gender, and healthcare cost data from claims data and (3) the model using both clinical data from the screening program and healthcare cost data calculated using claims data. Lastly, we developed the prediction models additionally including the data on diagnosis and procedure available in the claims data (21 major diagnosis categories and 22 major procedure categories) as predictors to investigate whether adding detailed claims data to our primary models improves the prediction performance.

A *P*-value of < 0.05 was considered statistically significant. All analyses were performed with R version 3.6.1. (The R Foundation for Statistical Computing). This study was a secondary data analysis of de-identified data (fully anonymized prior to receiving the data), and therefore, it was exempt from The University of California, Los Angeles Institutional Review Board review and participant consent was not required.

### Reporting summary

Further information on experimental design is available in the Nature Research Reporting Summary linked to this paper.

## Supplementary information

Supplementary Information

Reporting Summary

## Data Availability

The MinaCare data are the proprietary of MinaCare, Co., Ltd. and not publicly available for the research purpose. The researchers who would like to access the data for the research purpose should contact Dr. Yuji Yamamoto (mc_info@minacare.co.jp) in order to make a data use agreement and pay fee to have the data available.

## Code availability

Statistical codes and machine learning algorithms will be made available upon request submitted to the author.
