# Supplementary material for: Machine-learning-based prediction models for high-need high-cost patients using nationwide clinical and claims data
Source: NPJ Digit Med. 2020 Nov 11;3:148. doi: 10.1038/s41746-020-00354-8 (PMC7658979; doi:10.1038/s41746-020-00354-8)
Supplement: Supplementary file 1 — Supplementary Information [file 41746_2020_354_MOESM1_ESM.pdf]

**Supplementary information for:**

# Machine-Learning-Based Prediction Models for High-Need High-Cost Patients Using Nationwide Clinical and Claims Data

**Authors:**
Itsuki Osawa,[1] Tadahiro Goto,[2] Yuji Yamamoto,[3] Yusuke Tsugawa [4,5]

**Affiliations:**

1. Department of Medicine, The University of Tokyo Hospital, Tokyo, Japan
2. Department of Clinical Epidemiology and Health Economics, School of Public Health, The University of Tokyo, Tokyo, Japan.
3. MinaCare Co., Ltd., Tokyo, Japan
4. Division of General Internal Medicine and Health Service Research, David Geffen School of Medicine at UCLA, Los Angeles, CA, USA
5. Department of Health Policy and Management, UCLA Fielding School of Public Health, Los Angeles, CA, USA

**Table of Contents:**

Supplementary Table 1.

The variance inflation factor in the model for predicting HNHC patients p. 3

Supplementary Table 2.

Prediction ability of the reference and four machine-learning-based prediction models using additional 43 predictors (the diagnoses, procedures, etc.) from the claims data p. 4

Supplementary Table 3.

The list of variables in the national screening programs in Japan p. 5

Supplementary Table 4.

The rate of missingness and non-response of predictors in 36,316 people p. 6

**Supplementary Table 1. The variance inflation factor in the model for predicting HNHC patients**

| | sBP | dBP | FBS | HbA1c | TG | HDL-C | LDL-C | AST | ALT | y-GTP |
|---|---|---|---|---|---|---|---|---|---|---|
| VIF | 2.90 | 2.84 | 4.22 | 4.29 | 1.36 | 1.54 | 1.14 | 4.57 | 5.24 | 1.32 |

Abbreviations: VIF = Variance inflation factor; sBP = Systolic blood pressure; dBP = Diastolic blood pressure; FBS = Fasting blood sugar; HbA1c = Hemoglobin A1c; TG = Triglycerides; LDL-C = Low-density lipoprotein cholesterol; HDL-C = High-density lipoprotein cholesterol; AST = Aspartate aminotransferase; ALT = Alanine aminotransferase; y-GTP = Gamma-glutamyl transpeptidase

**Supplementary Table 2. Prediction ability of the reference and four machine-learning-based prediction models using additional 43 predictors (the diagnoses, procedures, etc.) from the claims data**

| Outcome | c-statistics | P-value** | Sensitivity | Specificity | PPV | NPV | PLR | NLR |
|---|---|---|---|---|---|---|---|---|
| Reference model* | 0.82 (0.81-0.83) | [Reference] | 0.66 (0.63-0.68) | 0.84 (0.84-0.85) | 0.15 (0.14-0.15) | 0.98 (0.98-0.99) | 4.2 (4.0-4.4) | 0.41 (0.38-0.44) |
| Logistic regression with Lasso regularization | 0.82 (0.81-0.83) | 0.96 | 0.66 (0.63-0.68) | 0.84 (0.84-0.85) | 0.15 (0.14-0.16) | 0.98 (0.98-0.99) | 4.2 (4.0-4.4) | 0.41 (0.38-0.44) |
| Random forest | 0.83 (0.82-0.85) | 0.18 | 0.68 (0.66-0.71) | 0.85 (0.84-0.85) | 0.15 (0.14-0.16) | 0.99 (0.98-0.99) | 4.4 (4.2-4.6) | 0.38 (0.35-0.41) |
| Gradient-boosted decision tree | 0.84 (0.82-0.85) | 0.08 | 0.67 (0.65-0.70) | 0.86 (0.85-0.86) | 0.16 (0.15-0.17) | 0.99 (0.98-0.99) | 4.6 (4.4-4.8) | 0.39 (0.36-0.42) |
| Deep neural network | 0.83 (0.82-0.84) | 0.22 | 0.69 (0.66-0.71) | 0.85 (0.84-0.85) | 0.15 (0.15-0.16) | 0.99 (0.98-0.99) | 4.5 (4.3-4.7) | 0.37 (0.34-0.40) |

Abbreviations: HNHC = High-Need, High-Cost; PPV = positive predictive value; NPV = negative predictive value; PLR = positive likelihood ratio; NLR = negative likelihood ratio

*We used a non-penalized logistic regression model as the reference model.

**We compared the area under the curve between each machine-learning-based prediction model and the logistic regression model (the reference model) using the DeLong's test.

**Supplementary Table 3. The list of variables in the national screening programs in Japan**

| Variables | <20 years | 20 years | | 25 years | | 30 years | | 35 years | | ≧40 years |
|---|---|---|---|---|---|---|---|---|---|---|
| Height | ✓ | * | * | * | * | * | * | * | * | * |
| Bodyweight | ✓ | ✓ | ✓ | ✓ | ✓ | ✓ | ✓ | ✓ | ✓ | ✓ |
| Waist circumference¶ | ✓ | ✓ | ✓ | ✓ | ✓ | ✓ | ✓ | ✓ | ✓ | ✓ |
| Visual examination | ✓ | ✓ | ✓ | ✓ | ✓ | ✓ | ✓ | ✓ | ✓ | ✓ |
| Auditory examination | ✓ | ✓ | ✓ | ✓ | ✓ | ✓ | ✓ | ✓ | ✓ | ✓ |
| ECG | * | * | * | * | * | * | * | ✓ | * | ✓ |
| Chest X-ray | * | ✓ | * | ✓ | * | ✓ | * | ✓ | * | ✓ |
| Sputum examination† | * | ✓ | * | ✓ | * | ✓ | * | ✓ | * | ✓ |
| Vital signs | | | | | | | | | | |
| Systolic blood pressure | ✓ | ✓ | ✓ | ✓ | ✓ | ✓ | ✓ | ✓ | ✓ | ✓ |
| Diastolic blood pressure | ✓ | ✓ | ✓ | ✓ | ✓ | ✓ | ✓ | ✓ | ✓ | ✓ |
| Laboratory data | | | | | | | | | | |
| RBC | * | * | * | * | * | * | * | ✓ | * | ✓ |
| Hb | * | * | * | * | * | * | * | ✓ | * | ✓ |
| Fasting blood sugar and/or HbA1c | * | * | * | * | * | * | * | ✓ | * | ✓ |
| TG | * | * | * | * | * | * | * | ✓ | * | ✓ |
| LDL-C | * | * | * | * | * | * | * | ✓ | * | ✓ |
| HDL-C | * | * | * | * | * | * | * | ✓ | * | ✓ |
| AST | * | * | * | * | * | * | * | ✓ | * | ✓ |
| ALT | * | * | * | * | * | * | * | ✓ | * | ✓ |
| y-GTP | * | * | * | * | * | * | * | ✓ | * | ✓ |
| Urinary sugar (qualitative analysis) | ✓ | ✓ | ✓ | ✓ | ✓ | ✓ | ✓ | ✓ | ✓ | ✓ |
| Urinary protein (qualitative analysis) | ✓ | ✓ | ✓ | ✓ | ✓ | ✓ | ✓ | ✓ | ✓ | ✓ |
| Survey responses | | | | | | | | | | |
| Past medical history and occupational history | ✓ | ✓ | ✓ | ✓ | ✓ | ✓ | ✓ | ✓ | ✓ | ✓ |
| Subjective and/or objective symptoms | ✓ | ✓ | ✓ | ✓ | ✓ | ✓ | ✓ | ✓ | ✓ | ✓ |

Abbreviations: ECG = Electrocardiogram; RBC = Red blood cell; Hb = Hemoglobin; HbA1c = Hemoglobin A1c; TG = Triglycerides; LDL-C = Low-density lipoprotein cholesterol; HDL-C = High-density lipoprotein cholesterol; AST = Aspartate aminotransferase; ALT = Alanine aminotransferase; y-GTP = Gamma-glutamyl transpeptidase.

✓: Required variables.

* : Optional variables.

¶ : Except for pregnant women.

† : Optional if a chest X-ray shows no abnormalities, including active or latent tuberculosis.

**Supplementary Table 4. The rate of missingness and non-response of predictors in 36,316 people**

| Variables | In 2013 | In 2014 | In 2015 | In 2016 |
|---|---|---|---|---|
| | Missing, n (%) | | | |
| Age | 0 (0) | 0 (0) | 0 (0) | - |
| Male gender | 0 (0) | 0 (0) | 0 (0) | - |
| Height | 49 (0.13) | 26 (0.07) | 45 (0.12) | - |
| Body weight | 45 (0.12) | 26 (0.07) | 47 (0.13) | - |
| Waist circumference | 5012 (14) | 4521(12) | 3916 (11) | - |
| Vital signs | | | | |
| Systolic blood pressure | 24 (0.07) | 51 (0.14) | 65 (0.18) | - |
| Diastolic blood pressure | 25 (0.07) | 51 (0.14) | 67 (0.18) | - |
| Laboratory data | | | | |
| Fasting blood sugar | 2729 (7.5) | 2267 (6.2) | 2732 (7.5) | - |
| HbA1c | 12941 (36) | 5115 (14) | 5775 (16) | - |
| TG | 103 (0.28) | 72 (0.20) | 116 (0.32) | - |
| LDL-C | 100 (0.28) | 71 (0.20) | 117 (0.32) | - |
| HDL-C | 105 (0.29) | 73 (0.20) | 117 (0.32) | - |
| AST | 99 (0.27) | 66 (0.18) | 198 (0.55) | - |
| ALT | 99 (0.27) | 66 (0.18) | 197 (0.54) | - |
| y-GTP | 98 (0.27) | 71 (0.20) | 198 (0.55) | - |
| Annual healthcare cost | 0 (0) | 0 (0) | 0 (0) | 0 (0) |
| | Non-response, n (%) | | | |
| ECG abnormalities | 35085 (97)* | 34572 (95)* | 34304 (94)* | - |
| Survey responses | | | | |
| Medications | | | | |
| Anti-hypertensive drugs | 5587 (15)* | 6328 (17)* | 6026 (17)* | - |
| Hypoglycemic drugs | 5604 (15)* | 6332 (17)* | 6028 (17)* | - |
| Anti-hyperlipidemic drugs | 5602 (15)* | 6328 (17)* | 6027 (17)* | - |
| Past medical history | | | | |
| Stroke | 19027 (52)* | 10772 (30)* | 10125 (28)* | - |
| Cardiovascular diseases | 19016 (52)* | 10764 (30)* | 10109 (28)* | - |
| Kidney diseases | 22404 (62)* | 14553 (40)* | 10120 (28)* | - |
| Current smoking | 3182 (8.8)* | 2754 (7.6)* | 2468 (6.8)* | - |
| Exercise > 30 mins twice a week | 16734 (46)* | 10392 (29)* | 9313 (26)* | - |

Abbreviations: HbA1c = Hemoglobin A1c; TG = Triglycerides; LDL-C = Low-density lipoprotein cholesterol; HDL-C = High-density lipoprotein cholesterol; AST = Aspartate aminotransferase; ALT = Alanine aminotransferase; y-GTP = Gamma-glutamyl transpeptidase; ECG = Electrocardiogram.
*All non-responded data were assumed to be normal (without any abnormalities) and other missing data substituted by the random forest method.
