# Supplementary material for: Machine-learning-based prediction models for high-need high-cost patients using nationwide clinical and claims data
Source: NPJ Digit Med. 2020 Nov 11;3:148. doi: 10.1038/s41746-020-00354-8 (PMC7658979; doi:10.1038/s41746-020-00354-8)
Supplement: Supplementary file 2 — Reporting Summary [file 41746_2020_354_MOESM2_ESM.pdf]

nature research

Corresponding author(s): Yusuke Tsugawa

Last updated by author(s): Sep 2, 2020

Reporting Summary

Nature Research wishes to improve the reproducibility of the work that we publish. This form provides structure for consistency and transparency in reporting. For further information on Nature Research policies, see our Editorial Policies and the Editorial Policy Checklist.

Statistics

For all statistical analyses, confirm that the following items are present in the figure legend, table legend, main text, or Methods section.

n/a	Confirmed	
☐	☒	The exact sample size (*n*) for each experimental group/condition, given as a discrete number and unit of measurement
☐	☒	A statement on whether measurements were taken from distinct samples or whether the same sample was measured repeatedly
☐	☒	The statistical test(s) used AND whether they are one- or two-sided<br>*Only common tests should be described solely by name; describe more complex techniques in the Methods section.*
☐	☒	A description of all covariates tested
☐	☒	A description of any assumptions or corrections, such as tests of normality and adjustment for multiple comparisons
☐	☒	A full description of the statistical parameters including central tendency (e.g. means) or other basic estimates (e.g. regression coefficient) AND variation (e.g. standard deviation) or associated estimates of uncertainty (e.g. confidence intervals)
☐	☒	For null hypothesis testing, the test statistic (e.g. *F*, *t*, *r*) with confidence intervals, effect sizes, degrees of freedom and *P* value noted<br>*Give P values as exact values whenever suitable.*
☒	☐	For Bayesian analysis, information on the choice of priors and Markov chain Monte Carlo settings
☒	☐	For hierarchical and complex designs, identification of the appropriate level for tests and full reporting of outcomes
☐	☒	Estimates of effect sizes (e.g. Cohen's *d*, Pearson's *r*), indicating how they were calculated

Our web collection on statistics for biologists contains articles on many of the points above.

Software and code

Policy information about availability of computer code

Data collection	We analyzed data from the national claims database from April 1, 2013, to March 31, 2016. The database collects claims from large employers and currently covers approximately 7.3% of the Japanese population.
Data analysis	All analyses were performed with R version 3.6.1. (The R Foundation for Statistical Computing). The packages we used were shown in Methods section.

For manuscripts utilizing custom algorithms or software that are central to the research but not yet described in published literature, software must be made available to editors and reviewers. We strongly encourage code deposition in a community repository (e.g. GitHub). See the Nature Research guidelines for submitting code & software for further information.

Data

Policy information about availability of data

All manuscripts must include a data availability statement. This statement should provide the following information, where applicable:

- Accession codes, unique identifiers, or web links for publicly available datasets
- A list of figures that have associated raw data
- A description of any restrictions on data availability

No additional data are available.

Field-specific reporting

Please select the one below that is the best fit for your research. If you are not sure, read the appropriate sections before making your selection.

☐ Life sciences ☒ Behavioural & social sciences ☐ Ecological, evolutionary & environmental sciences

For a reference copy of the document with all sections, see nature.com/documents/nr-reporting-summary-flat.pdf

Behavioural & social sciences study design

All studies must disclose on these points even when the disclosure is negative.

Study description	Prognostic study
Research sample	Our cohort from the claims data from large employers included all working individuals and their dependent family members, with a wide range of age groups.
Sampling strategy	We used a 10% random sample of 363,165 adults aged 18 years and older who underwent the national screening programs every year from 2013 through 2016 (approximately 45% of participants who received the screening in 2013 were included in our final sample).
Data collection	We collected the claims data from large employers, which currently cover approximately 7.3% of the Japanese population.
Timing	From April 1, 2013, to March 31, 2016.
Data exclusions	Not applicable
Non-participation	Not applicable
Randomization	Not applicable

Reporting for specific materials, systems and methods

We require information from authors about some types of materials, experimental systems and methods used in many studies. Here, indicate whether each material, system or method listed is relevant to your study. If you are not sure if a list item applies to your research, read the appropriate section before selecting a response.

Materials & experimental systems

n/a	Involved in the study	
☒	☐	Antibodies
☒	☐	Eukaryotic cell lines
☒	☐	Palaeontology and archaeology
☒	☐	Animals and other organisms
☒	☐	Human research participants
☐	☒	Clinical data
☒	☐	Dual use research of concern

Methods

n/a	Involved in the study	
☒	☐	ChIP-seq
☒	☐	Flow cytometry
☒	☐	MRI-based neuroimaging

Clinical data

Policy information about clinical studies

All manuscripts should comply with the ICMJE guidelines for publication of clinical research and a completed CONSORT checklist must be included with all submissions.

Clinical trial registration	Not applicable.
Study protocol	Prognostic study of claims data from the large employers from April 1, 2013, to March 31, 2016.
Data collection	We analyzed data from the national claims database from April 1, 2013, to March 31, 2016. The database collects claims from large employers and currently covers approximately 7.3% of the Japanese population.
Outcomes	The outcome was becoming an High-need, high-cost (HNHC) patient in the subsequent year. We defined HNHC patients as those who account for the top 5% of annual healthcare costs.
